# Advances in Nano-Enabled Platforms for the Treatment of Depression

**DOI:** 10.3390/polym13091431

**Published:** 2021-04-29

**Authors:** Fadzai P. Mutingwende, Pierre P. D. Kondiah, Philemon Ubanako, Thashree Marimuthu, Yahya E. Choonara

**Affiliations:** Wits Advanced Drug Delivery Platform Research Unit, Department of Pharmacy and Pharmacology, School of Therapeutic Sciences, University of Witwatersrand, 7 York Road, Parktown, Johannesburg 2193, South Africa; fmutingwende@gmail.com (F.P.M.); pierre.kondiah@wits.ac.za (P.P.D.K.); philemon.ubanako@wits.ac.za (P.U.); thashree.marimuthu@wits.ac.za (T.M.)

**Keywords:** drug delivery, antidepressants, biopolymers, nanocarriers, nanomedicines, biomedical nanotechnology

## Abstract

Nanotechnology has aided in the advancement of drug delivery for the treatment of several neurological disorders including depression. Depression is a relatively common mental disorder which is characterized by a severe imbalance of neurotransmitters. Several current therapeutic regimens against depression display drawbacks which include low bioavailability, delayed therapeutic outcome, undesirable side effects and drug toxicity due to high doses. The blood–brain barrier limits the entry of the drugs into the brain matrix, resulting in low bioavailability and tissue damage due to drug accumulation. Due to their size and physico-chemical properties, nanotechnological drug delivery systems present a promising strategy to enhance the delivery of nanomedicines into the brain matrix, thereby improving bioavailability and limiting toxicity. Furthermore, ligand-complexed nanocarriers can improve drug specificity and antidepressant efficacy and reduce drug toxicity. Biopolymers and nanocarriers can also be employed to enhance controlled drug release and reduce the hepatic first-pass effect, hence reducing the dosing frequency. This manuscript reviews recent advances in different biopolymers, such as polysaccharides and other nanocarriers, for targeted antidepressant drug delivery to the brain. It probes nano-based strategies that can be employed to enhance the therapeutic efficacy of antidepressants through the oral, intranasal, and parenteral routes of administration.

## 1. Introduction

Depression is a common mental disorder that is characterized by a persistent feeling of sadness, low self-esteem, disturbed appetite, suicidal thoughts, insomnia and loss of interest [1]. Depression is caused by several aspects which include pathological effects, social activities such as drug and alcohol abuse and biological factors [2]. According to research done by the World Health Organization (W.H.O) in 2017, more than 300 million people (approximately 4.4% of the world’s population) suffer from depression [1] making it one of the top two causes of disability-adjusted life years currently [2]. Pathological causes of depression include a chemical imbalance in the brain, energy metabolic decline and alteration in body hormones [3]. According to the serotonin hypothesis, depression is a result of dysfunctional serotonergic activities [4] which results in reduced serotonin levels in the brain. Several classes of antidepressant therapy that are currently on the market include selective serotonin reuptake inhibitors (SSRI), tricyclic antidepressants, serotonin-norepinephrine reuptake inhibitors, and monoamine oxidase inhibitors. SSRI such as paroxetine, vilazodone, and fluvoxamine are first-line treatment options in adults with depression, albeit with several contraindications [5]. The side effects of current medication include delayed therapeutic onset, low bioavailability, erectile dysfunction, weight gain, dry mouth, nervousness, and insomnia. Some currently approved antidepressant drugs pass through extensive first-pass metabolism which results in reduced oral bioavailability [5]. The time taken by the drug to reach the saturation point is usually prolonged, resulting in delayed therapeutic onset and reduced therapeutic efficacy. Furthermore, as the bioavailability is low, higher doses are required, leading to an increased prevalence of side effects. The therapeutic effect is also limited because of the presence of the blood-brain barrier (BBB) and the blood–cerebrospinal fluid barrier (BCSFB). Traditional medicines have a limited capacity of crossing the BBB and BCSFB [6]. According to current research, nanotechnology-based delivery platforms can be employed to ameliorate the above-mentioned limitations [7,8]. The uses of nanomedicine, biopolymers and nanocarriers have gained significant attention on overcoming these gaps [7].

Nano-based drug delivery strategies offer various advantages in the treatment of chronic diseases by site-specific and targeted delivery, thereby improving the efficacy of approved formulations [7]. Additionally, nanoparticles can improve plasma bioavailability profiles, further enhancing a sustained delivery of antidepressants, resulting in reduced side effects on account of lowered dosing frequencies. Nanomedicine has been used to overcome the limitations of the BBB, as they penetrate through it due to their small size ≤100 nm [9]. Furthermore, nanoparticles can target specific receptors enabled by complexation to ligands such as transferrin and glutathione for improving therapeutic efficacy [9,10,11]. In this review, we discuss different drug carriers, ligands and biopolymers that can improve the bioavailability and therapeutic efficacy of antidepressants by reducing undesirable side effects and dosing frequencies, to achieve safe, desired clinical outcomes. 

## 2. Designing Sustained-Release Formulations for the Delivery of Antidepressant Drugs

Biopolymer-based nano-drug delivery systems have been shown to lower antidepressant drug toxicity and dosing frequency, as they can be designed to exhibit controlled drug release [12]. According to literature, drug solubility, specificity, improved bioavailability, bio-distribution and controlled release of antidepressant drugs have been achieved through the use of biopolymer-based drug delivery [12]. Nanocarriers influence the bioavailability and therapeutic efficacy of antidepressants depending on the route of administration used. They can enhance the drug stability, encapsulating rate, drug solubility, and circulation time of antidepressants in the body [13]. 

### 2.1. The Oral Route of Antidepressant Drug Delivery

The oral route of administration is the route of choice for the delivery of chronic therapy due to good patient compliance, low cost and ease of administration. However, enzymatic barriers in the gastrointestinal tract harm the effectiveness of oral drug delivery. The oral route of delivery limits drug half-life due to the hepatic first-pass effect and other factors, resulting in reduced drug efficacy [14,15]. The BBB is a complex physiological barrier, which hinders the penetration of substances into the brain. To improve neuro-availability, biopolymers can be employed to improve drug permeability through the BBB. Biopolymers such as polysaccharides present attractive strategies for the oral delivery of antidepressants due to their desired characteristics which include; sustained drug release profiles, small size, stability, biodegradability, biocompatibility and limited toxicity [16].

**Figure 1:**
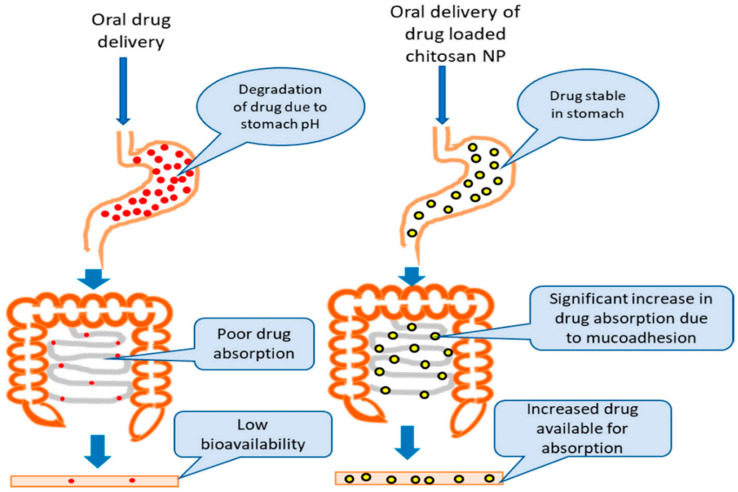
Chitosan improves drug oral bioavailability. In vivo comparison of oral bioavailability of a chitosan-based nanoparticle and pure drug to investigate the effect of chitosan-based nanoparticles on improving the drug availability for absorption via the intestinal epithelium. Adapted and modified with permission from [21]. Copyright (2019) MDPI.

A study was conducted in which venlafaxine was delivered in a copolymeric platform composed of sodium alginate and hydroxypropyl methylcellulose. The main focus of the study was to improve the oral bioavailability of venlafaxine as it is freely soluble in water, undergoes first-pass metabolism, has a narrow absorption window, and exhibits a short half-life [14]. The results showed a sustained release profile as well as improved oral bioavailability and mucoadhesive properties of the antidepressant, resulting in improved drug efficacy [14]. The drug diffusion release in the above-mentioned study was dependent on the swelling behavior of the biopolymer and the concentration of sodium alginate employed. A high concentration of sodium alginate resulted in controlled release hence improved half-life [14]. Another study showed that sodium alginate delays gastric emptying, hence increased drug circulation time when imipramine was encapsulated into sodium alginate-based nanoparticles for oral delivery [17]. Moreover, using chitosan to improve the oral bioavailability of escitalopram demonstrated reduced first-pass metabolism, as illustrated in Figure 1 [18]. The results showed reduced hepatic first-pass metabolism, with shielding from enzymatic degradation. The in vitro study which was done using a dialysis membrane exhibited improved drug circulating time in the body, hence sustained release over 24 h. The oral bioavailability of the antidepressant was improved due to the mucoadhesive properties of the biopolymer. The drug release was 98.4 ± 1.07%, the particle size ranged from 60–115 nm and the neuro- availability of the antidepressant was improved. The size and biodegradability properties of chitosan affect its bioactivity on the BBB. Hence, drug-loaded chitosan nanoparticles can penetrate through the BBB for drug delivery against neurological disorders.

Chitosan nanoparticles are also biocompatible, biodegradable, mucoadhesive and nontoxic, which makes them suitable for designing drug delivery vehicles for the treatment of neurological conditions [18,19]. Erum et al., 2016, formulated and evaluated fluoxetine HCL microspheres using chitosan and arabinoxylan as the biopolymers. These biopolymers are known to be non-toxic, biocompatible, biodegradable, and have improved entrapment efficiency. The cationic property of chitosan allows it to react with polyanions giving rise to polyelectrolyte complexes with arabinoxylan biopolymers. The percentage yield was improved to 93% and the drug entrapment rate was improved to about 77%. In vitro studies proved that chitosan improved the drug release of the antidepressant due to swelling properties of the biopolymers. Hence, a copolymer of chitosan and arabinoxylan can be employed during the formulation of fluoxetine HCL oral microspheres in a 2:1 ratio, respectively, to obtain a sustained release profile. Moreover, chitosan has a high drug entrapping efficiency and the percentage yield is directly proportional to the concentration of the biopolymers [20]. This might result in improved neuro-bioavailability of the antidepressants.

**Figure 2:**
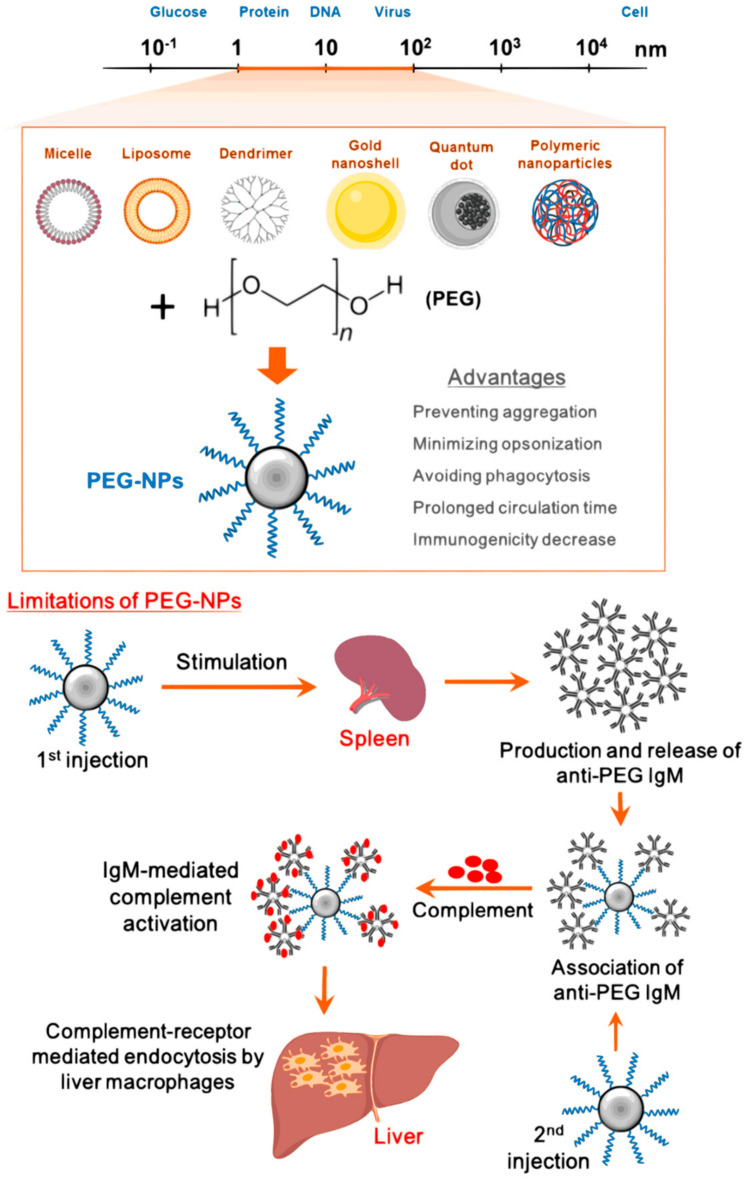
Effect of PEGylation. Polyethylene glycol (PEG) is conjugated to nanoparticles (NPs) surface to form PEG-NPs, providing several advantageous properties within a drug delivery system. The diagram also indicates the limitations associated with PEGylating of nanoparticles. Adapted and modified with permission from [22]. Copyright (2020) MDPI.

Moreover, PEGylated oral nanoparticles exhibit enhanced half-life, oral bioavailability, and water solubility of bioactive molecules, reduced hepatic first-pass metabolism and premature leaking of the bioactive agent. PEGylation also increases the molecular weight of the drug, hence slowing down its clearance rate in the kidney. Figure 2 summarizes the properties of PEGylated nanoparticles [22,23]. PEGylated polymeric nanoparticles penetrate the brain better, as compared to nanoparticles coated with polysorbate 80 [24]. This is because PEG is covalently attached to the polymer, preventing the pre-leakage of the bioactive agent from the PEGylated polymeric nanoparticles whilst polysorbate 80 is prone to adsorption [24].

Abourehab et al. conducted a study where they synthesized self-assembled biodegradable polymeric micelles to improve dapoxetine-loaded PEG-PLGA delivery of the antidepressant nanomicelles across the BBB. The study showed that PEGylation might protect the drug-loaded nanoparticle from uptake by the reticulo-endothelial system, hence improving the oral bioavailability and the circulation half-life of the drug. The extended circulation half-life allows more absorption of loaded PEG-PLGA via the bloodstream. The study further demonstrated that PEGylation of the nanoparticles improves the rate of accumulation of the antidepressant in the brain and its absorption rate on account of reduced first-pass metabolism. Furthermore, the improved half-life ameliorates the accumulation of the antidepressant, hence increasing its cerebral concentration [25].

### 2.2. Intranasal Route of Administration of Antidepressants

The intranasal route of administration has gained momentum in enhancing the efficacy of antidepressants due to diminished first-pass metabolism, circumvention of the BBB and non-invasiveness [26,27]. The intranasal route transports drugs to the CNS through the olfactory and trigeminal nerve pathways [28]. Poly(lactic-*co*-glycolic acid-Chitosan (PLGA-CN) nanoparticles possess biocompatible, mucoadhesive, bio-adhesive and biodegradable properties which support the prolonged circulation of the loaded nanoparticles in the nasal cavity and reduced nasal mucociliary clearance. To investigate the mucoadhesive properties of desvenlafaxine (DVF) loaded PLGA-CN nanoparticles on the brain, Tong et al. conducted a study in which they incorporated DVF into a CN and PLGA copolymeric platform. The in vitro studies displayed a sustained release profile for over 24 h. When PLGA-CN nanoparticle loaded with DVF were intranasally administered in in vivo depression rat animal models, the bioavailability was improved (56.35%) when compared with the bioavailability of the free drug (23.70%). Chitosan possesses the ability to enhance paracellular transport through epithelial tight junctions due to its interaction with the protein kinase C pathway in mucosal epithelial cells. The findings demonstrated that the entrapping efficacy, neuro-bioavailability, and uptake of the antidepressant into the brain, and efficacy were improved. Moreover, DVF/PLGA-CN nanoparticles displayed improved brain targeting efficacy and uptake of DVF by the brain [27]. Chitosan biopolymer lowers the rate of mucociliary clearance and opens the tight junctions between cells rapidly, which facilitates drug transport across the nasal membrane to the brain by the paracellular route. The small size of chitosan nanoparticles improves their transport across the nasal mucosa. In a previous study, venlafaxine-loaded chitosan nanoparticles were formulated to investigate the ability of venlafaxine-loaded chitosan nanoparticles to enhance drug delivery to the brain via intranasal administration, thereby improving the treatment of depression. The ex vivo studies that were carried out using a Franz diffusion cell on the porcine nasal mucosa, resulted in enhanced uptake of venlafaxine-loaded chitosan nanoparticles by three-fold, relative to the free venlafaxine solution.

The in vivo studies that were conducted in rat animal models resulted in increased bioavailability. The data showed that the intranasal route of administration can improve the uptake of venlafaxine-loaded chitosan nanoparticles by the brain [29]. Venlafaxine-loaded alginate nanoparticles have also been synthesized and characterized for the nasal route of administration [30]. When the particles were analyzed in vitro, they displayed a sustained release profile over a period of 24 h. The ex vivo studies that were done using porcine nasal mucosa showed that venlafaxine-loaded alginate nanoparticles significantly increased the permeation of venlafaxine as well as the mucosal absorption rate. The in vivo studies demonstrated that the mucoadhesive properties of venlafaxine/alginate nanoparticles also improved the concentration of the venlafaxine in the brain. The researchers concluded that alginate-loaded nanoparticles can improve the therapeutic effect of antidepressants [30]. Furthermore, venlafaxine-loaded alginate nanogels have been formulated and characterized for the treatment of depression. The formulated venlafaxine-loaded nanogel was stable at pH 5.4 ± 0.3 and also in the nasal cavity. The efficacy and safety of the venlafaxine-loaded nanogel were investigated using sheep nasal mucosa membrane. The drug accumulation release was found to be 96.96% ± 0.13%, and there was no evidence of tissue damage. The improved permeation of the antidepressant was attributable to the interaction of negatively charged sialic acid residues on the cell membrane and tight junctions of the mucosal epithelial cells and positively charged amino group on the alginate biopolymer resulting in the opening of tight junctions. The in vivo studies conducted with rats indicated an improved concentration of the drug in the brain, hence improved efficacy of the antidepressant. This study showed that venlafaxine-loaded nanogels can improve the drug delivery of antidepressants and are safe for intranasal administration [31]. However, clinical studies need to be done to prove the safety and efficacy of the delivery system.

### 2.3. Parenteral Route for the Delivery of Antidepressants

The parenteral route of drug delivery possesses attractive attributes such as the avoidance of hepatic first-pass metabolism, improved bioavailability and reliable doses; thereby enhancing the efficacy of antidepressants [32]. For example, sertraline-loaded chitosan nanoparticles have been formulated and characterized for the treatment of depression [21,33]. A single dose of sertraline nanoparticles was intravenously administered into a rabbit via the marginal ear vein. The half-life and the entrapment rate of the sertraline nanoparticles were improved. The plasma bioavailability of the loaded nanoparticles quadrupled when compared with the pure drug on account of the mucoadhesive properties of the chitosan. The data showed that chitosan-loaded nanoparticles prolonged the circulation period of sertraline and enhanced its plasma bioavailability [33]. L–tyrosine-loaded nanoparticles have also been synthesized, characterized and administered to rat models for the treatment of depression. The size of the loaded nanoparticles was found to be ±141.8 nm and the entrapping rate was 87.45%. The in vitro studies of L-tyrosine-loaded nanoparticles also showed a sustained release profile of ±86.65% over 48 h. The study proved the safety of the nanoparticles an improved drug efficacy of ±86.65%. The data indicated that the parenteral administration of L-tyrosine-loaded nanoparticles improves its efficacy [34]. The study did not report any toxicity caused by the nanoparticles, prompting the need for further clinical and in vivo studies to be conducted. Summary of biopolymers discussed is presented in Table 1.

## 3. Nanocarriers Employed as Therapeutic Delivery Platforms of Antidepressants

Nanocarriers possess attractive properties which include a high surface-area-to-volume ratio, controlled drug release, targeted delivery, multi-functionality, and a great potential for surface modification [35,36]. Moreover, their nano size has conferred on them the ability to penetrate the BBB and target the brain, rendering them desirable for neurotherapy and diagnosis. Nanocarriers can be employed to enhance drug solubility, circulating time, stability, and the biocompatibility of antidepressants targeted to the brain [35,37]. Moreover, nanocarriers minimize hepatic first-pass metabolism and protect bioactive agents from hydrolytic and enzymatic degradation [38]. They show great potential in improving antidepressant drug delivery due to their characteristics [35]. The use of nanocarriers to improve the efficacy of delivery systems of antidepressants has gained increased attention among researchers [35].

### 3.1. Dendrimers

Dendrimers are nano-sized artificial macromolecules with monodispersed structures and hyperbranched synthetic polymer systems [39]. Dendrimers have garnered significant interest from researchers as drug carriers for several neurological disorders due to their attractive properties which include increased half-life, rapid cellular entry, high drug loading capacity, improved delivery efficiency, biocompatibility, targeting ability, stability, and reduced side effects [12,40]. Furthermore, they can be used to deliver both hydrophobic and hydrophilic drug molecules and can maintain drug levels in a therapeutically desired range [12]. Dendrimers can be modified with linkages and conjugated with specific ligands to improve biocompatibility and enhance targeted delivery to the CNS. In a previous study, poly(amidoamine) dendrimers crosslinked with PEG hydrogel was used as a nanocarrier for the antidepressant, venlafaxine [41]. The in vitro results displayed sustained drug release of the antidepressant due to the swelling properties of the nanocarrier, and reduced drug toxicity due to a decreased dosing frequency. The data indicated that the incorporation of PEG hydrogel improved the sustained release profile of the drug and stability of the nanoparticles [41].

### 3.2. Nanogels

**Figure 3:**
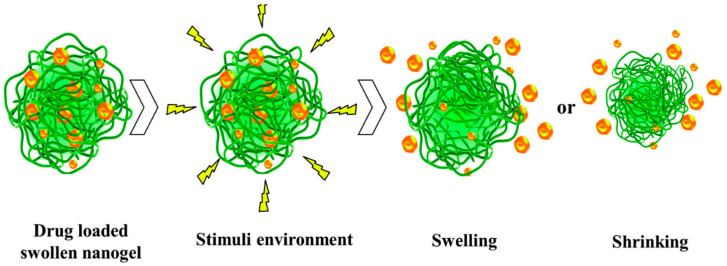
Drug release from a nanogel network under stimuli environments. The swelling or shrinking process of nanogel under stimuli environment to attain the controlled release. Adapted from [44] Copyright (2017) Taylor and Francis.

Nanogels are three-dimensional nanoscale hydrogel materials that are formed by chemically or physically crosslinking, hydrophilic or amphiphilic polymer networks. Nanogels have a high capacity of retaining water without being dissolved or denatured, thereby maintaining an intact structure [36,42]. They have a large surface area, protect bioactive agents from premature leakage, and can be employed to deliver bioactive agents which includes antidepressants in a controlled release manner when stimulated (Figure 3) [43,44]. Nanogels possess desirable properties for the delivery of antidepressants. They are biodegradable, non-immunogenic, have a high entrapping rate, drug loading capacity, permeability and are highly biocompatible due to their hydrophilic features [42]. The size of nanogels allows them to penetrate the smallest capillary vessels, hence improving their circulation in the blood and thereby enhancing the bioavailability of the contained drug [42]. The drug release mechanism from nanogels involves degradation of the nanogel structure, simple diffusion, and pH- or temperature-induced changes. Nanogels can improve the delivery of antidepressants because their properties can be altered to deliver drugs at targeted sites; leading to diminished side effects and enhanced therapeutic outcome [42,45].

A few studies have evaluated the effectiveness of nanogels as delivery systems for antidepressant drugs. In one study, formulated venlafaxine-loaded nanogels showed an improved drug encapsulation of 88% ± 4.163%. The in vitro analysis used to investigate the drug release displayed a sustained release profile [31]. Moreover, the nanogel displayed a rapid onset with a long duration of action compared to the pure drug solution. The formulation showed good stability with particle size and zeta potential of 150 nm and −8.08 mV, respectively [31]. The ex vivo studies indicated that the permeation rate of the venlafaxine-loaded nanogel had improved [31]. In another study, paroxetine- and duloxetine-loaded nanogels were formulated to enhance their drug release profiles. The in vitro studies of the loaded nanogels displayed a sustained release profile with duloxetine’s release profile higher by 10%. The study proved that biocompatible nanogels can be used to design formulations for the sustained release of antidepressants and have the potential of maintaining long-term antidepressant activity [46].

### 3.3. Polymeric Micelles

Polymeric micelles are nanocarriers that are formed by self-association of amphiphilic block copolymers in aqueous solutions [47]. They can be used to deliver oral antidepressants which are poorly water-soluble. Polymeric micelles possess important properties that can improve the aqueous solubility, stability, bioavailability and half-life of the oral antidepressants [48]. Furthermore, micelles have other properties which include controlled delivery of hydrophobic drugs, target specificity, low toxicity, biodegradability, biocompatibility and their nano size [49,50]. They also display a slow rate of dissociation which increases the retention time of the loaded drug. The hydrophilic shell stabilizes and supports the hydrophobic core in the aqueous medium hence improving the solubility of the biopolymer in the medium, while the hydrophobic core protects the drug [49]. Moreover, micelles can protect the drug from interfering with serum proteins, non-targeted cells, harsh conditions of the gastrointestinal tract (GIT) and facilitate safe transportation through the GIT. Nanomicelles also improve drug absorption through the GIT mucosa giving credit to their enhanced permeability. Due to these properties, micelles can be used to deliver drugs to the brain using non-toxic polymers. According to another study, polymeric micelles present an attractive potential for enhancing the sustained release of antidepressants.

Polymeric micelles can also enhance the permeability of the BBB through copolymer interaction with cell membranes that improve membrane fluidity, inhibit P-glycoprotein and multidrug efflux transporters. In the above study, ex vivo studies were carried out on bovine intestines, while rat animal models were used for in vivo studies to investigate the delivery of dapoxetine in a polymeric nano-micelle across the BBB. The ex vivo studies displayed that the permeation rate was found to be 91.27% ± 7.64%. Brain cells from three rats used for the investigation showed that polymeric micelles loaded with the antidepressant displayed high kinetic stability, improved drug solubility and oral bioavailability of the encapsulated dapoxetine by 2.7 folds [25]. The results indicated that polymeric micelles enhanced the distribution of dapoxetine into the brain matrix and reduced its elimination rate due to a delay in residency time. The data suggested that dapoxetine-loaded polymeric micelle formulations improved both delivery across the BBB and oral bioavailability of the drug [25].

However, polymeric micelles possess drawbacks which include, low drug loading capacity and poor drug release from the nanomicelles if the drug particles are too large [9]. They are also prone to premature drug leaking due to low drug incorporation stability, which might decrease drug bioavailability. Furthermore, their ability to show controlled release requires certain proprieties such as low chain mobility core and high thermodynamic and high kinetic stability in a viscous medium [51]. Drugs with a high diffusion coefficient are unsuitable for incorporation into nanomicelles as they tend to display an immediate release and not the desired sustained release profiles [51].

### 3.4. Nanoliposomes

Nanoliposomes can be defined as nanoscale bilayer lipid vesicles. They can improve drug permeability through the BBB hence a high concentration of antidepressants can be delivered considering that the BBB is highly selective [52]. Nanoliposomes are made up of phospholipids with an aqueous reservoir which gives them the ability to have a high encapsulation rate [53]. The nanoparticle lipid bilayer is compatible with the lipid layer of the BBB because both layers are similar physiological membranes. The similarity in both membranes confers a positive impact on the BBB permeability of the drug. Nanoliposomes are compatible with both hydrophobic and hydrophilic drug molecules. Besides, they show several characteristics which make them good drug carrier systems for CNS conditions which include biodegradability, biocompatibility, improved intracellular uptake and solubility of the bioactive agents, and reduced toxicity [54,55]. Nanoliposomes can impart controlled drug release resulting in improved therapeutic efficacy and reduced side effects. Moreover, they reduce the rate of first-pass effect in the liver [53]. Nanoliposomes can also be complexed with ligands to improve the specificity; thereby resulting in enhanced bioavailability and reduced undesirable side effects. They have been shown to protect bioactive agents from degradation, hence increasing oral bioavailability. According to the literature, nanoliposomes have successfully improved the oral bioavailability of various compounds such as lipophilic and hydrophobic bioactive agents [56]. Notwithstanding, nanoliposome carrier systems display several limitations such as poor stability in aqueous environments due to their mechanical structure, high cell-penetrating ability, and increased chances of serum protein binding. Considering that the nanoliposomes have poor stability under physiological conditions, oral drug delivery would also be complicated [53].

To our best knowledge, no research has been published on the use of nanoliposomes as nanocarriers for the delivery of antidepressant therapy. However, nanoliposomes have been used for the delivery of drugs in other neurological conditions such as Alzheimer’s disease. Alzheimer’s disease is a neurodegenerative disease characterized by the accumulation of toxic proteins in the brain [57]. According to a study that was done, nanoliposomes can increase the penetration of rivastigmine through the BBB. The in vivo studies proved that the nanoliposomes have the potential of protecting the drug from the enzymatic and pH degradation, hence increasing the therapeutic efficacy. The ex vivo studies that were done using the Madin-Darby Canine Kidney (MDCK) cell line showed improved permeation of the drug [58]. Rotman et al. synthesized glutathione PEGylated liposome for the delivery of anti-amyloid antibodies against Alzheimer’s disease. The bioavailability of the antibody and target specificity was improved because of size and surface modification of the nanoliposomes. The in vivo studies that were conducted using mouse animal models proved that nanoliposomes can cross the BBB and they can be retained for a longer period, enhancing the neurological bioavailability [59].

### 3.5. Carbon Nanotubes (CNT) 

Carbon nanotubes are molecules that comprise a single sheet of carbon atoms rolled up into a cylindrical shape. CNTs possess chemical and structural properties that render them good drug carrier systems for drug delivery to the CNS [60,61]. CNTs show high biocompatibility and solubility which are determined by certain parameters that include size, physical properties and morphology of the modified molecules. These parameters determine the therapeutic outcome as they affect the biocompatibility of the molecule with the body [6]. CNTs can entrap high drug volumes owing it to their spherical shape and high surface area to volume ratio [62]. They also shield the drug from degradation during transportation and release it either through a chemically- or electrically controlled release. CNTs have low solubility several in solvents compatible with the biological milieu and it is hard to maintain high quality with negligible impurities [63]. CNTs permeability into the brain cells is dependent on temperature; with higher temperatures leading to decreased permeability [9]. To our best knowledge, CNTs have not yet been investigated as nanocarriers for antidepressants delivery. Notwithstanding, they have been researched for other neurological conditions such as Alzheimer’s and Parkinson’s diseases [64]. In one study, single-walled nanotubes were synthesized for the targeted delivery of dopamine into the brain of parkinsonian mice [64].

Parkinson’s disease is a neurodegenerative disorder in which there will be low levels of dopamine in the brain. The study aimed to improve the permeation of dopamine, target delivery and to improve neurological bioavailability. PEGylation of carbon nanotubes improved feasibility and therapeutic efficacy of dopamine. PC 12 cell line was used for ex vivo analysis. PC 12 cell line was used due to its properties which include the ability to take up and release dopamine. The ex vivo proved that the carbon nanotubes have a potential of enhancing the permeation of dopamine due to their size of less 200 nm and surface modification. The pre-clinical study showed that small doses of carbon nanotubes (25 mg/kg) are safe for delivery in parkinsonian mice when using the parental route of administration [64].

### 3.6. Solid-Lipid Nanoparticles (SLN) 

**Figure 4:**
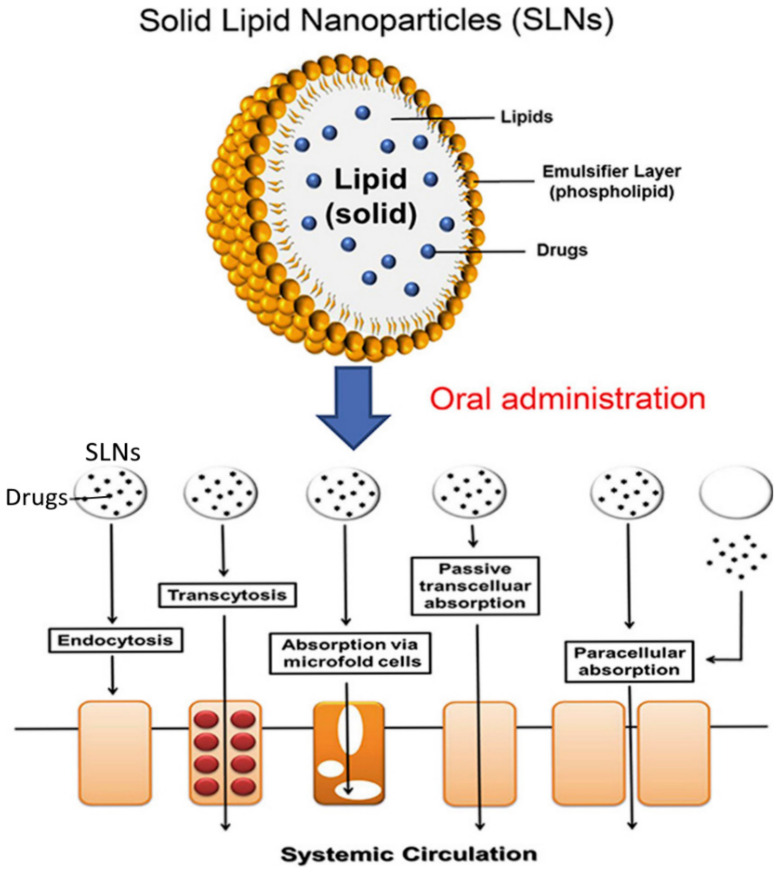
Mechanism of oral solid lipid nanoparticles (SLN) using different routes of delivery. Encapsulation of the lipophilic moiety of phospholipids in the lipid matrix and the absorption of drugs across the gastrointestinal tract. Adapted and modified with permission from [15] Copyright (2017) Science Direct.

SLNs are lipid-based and can overcome the limitations exhibited by the other colloidal carriers, due to good physical stability and excellent drug release profiles [65]. Moreover, SLNs are biodegradable, easy to synthesize, non-toxic and display controlled release properties. Due to their attractive characteristics, SLNs possess the potential to improve the efficacy of antidepressant drug delivery. SLNs display enhanced stability, improved bioavailability, improved epithelial permeability, prolonged half-life, enhanced permeability through the BBB and reduced toxicity [15,66,67]. Furthermore, SLNs can be used to deliver both hydrophilic and lipophilic drugs, making them versatile drug delivery vehicles. They also have a large surface area due to their nano-sized feature, resulting in an improved absorption rate. The physicochemical properties of SLNs such as surface charge, size, lipophilicity and surface property can be modified to enhance the penetration of SLNs across the gastrointestinal membrane (see Figure 4). They also improve the oral bioavailability of drug molecules due to decrease in hepatic first-pass effect through the use of emulsifiers [15]. Several studies have shown that SLNs could increase the oral bioavailability and therapeutic efficacy of antidepressants [15]. Venlafaxine is a substrate of P-glycoprotein with lowered permeability through gastrointestinal and BBB. In one study, venlafaxine-loaded SLNs administered to mice via the oral route demonstrated a 1.5 fold higher concentration of the drug from SLNs in the brain and plasma when compared with venlafaxine alone. This data proved that SLNs can enhance the oral bioavailability of venlafaxine and its accumulation in the brain [15]. The SLNs also showed reduced P-glycoprotein-mediated efflux of venlafaxine, hence improving the penetration of the venlafaxine-loaded SLNs through the BBB [15]. Moreover, an in vivo study using mice indicated that SLN nanocarriers enhance the oral uptake of antidepressants by accessing the lymphatic system, hence improving oral bioavailability [68]. Overall, the data showed that SLNs can be used to improve the efficacy of antidepressants [68]. In another research study where the antidepressant, duloxetine was encapsulated in SLNs, the oral bioavailability of the drug was improved owing to reduced first-pass metabolism of the duloxetine-SLN system when compared with duloxetine only [69]. The drug–nanoparticle formulation was stable under acidic media and it displayed improved pharmacological properties in vivo. The in vivo studies that were done using mice proved that the SLN enhanced the release profile and neuro-bioavailability of the antidepressant. The nanoparticles also displayed a sustained release profile in in vitro [69].

Currently, no SLNs have been clinically approved as drug carriers for CNS conditions. Although many in vitro and preclinical studies have been carried out on SLN-mediated drug delivery, clinical trials are still limited [15]. The paucity of clinical trials on SLNs might be due to insufficient in vitro and preclinical data to prove their efficacy and biocompatibility. On the negative side, SLNs show lipid particle growth, are prone to gelation and have a poor incorporation rate which can be affected by the molecular weight of the types of compounds involved [70]. The loading capacity of SLNs can either be improved or decreased by the length of the hydrocarbon chain, depending on the physico-chemical properties of the drug. This might result in low oral bioavailability if the entrapment efficiency is low. The stability and specificity can also be affected by lipids, surfactants and co-surfactant used [71]. In other cases, the diseases or condition might become under-treated because the drug molecule is released very slowly. Sometimes, the drug molecule delays accumulating in the targeted organ due to prolonged drug circulation in the body [15].

### 3.7. Polymeric Nanoparticles

Polymeric nanoparticles are sub-micron particles composed of active pharmaceutical substances encapsulated within or adsorbed onto polymers [72]. Due to their nano size, they have a high potential of being taken up by cells and they can penetrate blood capillaries. This leads to improved bioavailability as a result of an increased rate of drug accumulation at the target organs. The specificity of antidepressants can be amended by conjugating a ligand covalently to the polymeric nanoparticles [23]. As a drug carrier, it displays sustained drug release, biodegradability, prolonged duration, ability to deliver peptides, proteins, and genes through the oral route of administration, and high stability during storage [23,73]. Moreover, polymeric nanoparticles can cross the BBB via receptor-mediated endocytosis [74]. In one study where the antidepressant effect of l-tyrosine-loaded polymeric nanoparticles was investigated, enhanced therapeutic efficacy and drug safety were observed [34]. Another study demonstrated that when desvenlafaxine was encapsulated in PLGA-CN-loaded polymeric nanoparticles the mucoadhesive properties and the retention time of the antidepressant in the nasal cavity were increased; thereby improving the circulation time of the drug [27]. Moreover, encapsulated escitalopram in polymeric nanoparticles composed of chitosan and tripolyphosphate biopolymers have been used to enhance the drug release profile. The in vitro study conducted using a dialysis membrane displayed a sustained release profile of up to 98.4% drug release from the loaded polymeric nanoparticles and about 78.6% for the pure drug over a period of 24 h. The encapsulation rate of the antidepressant was improved to about 79%. The researchers concluded that polymeric nanoparticles can be used for the sustained drug release of antidepressants [18].

### 3.8. Magnetic Nanoparticles 

Magnetic nanoparticles (MNPs) are generally spherical and crystalline nanoparticles that are composed of elements with unpaired electrons such as iron (Fe), nickel (Ni) and chromium (Cr) which confer magnetic properties on them. Their magnetic properties are harnessed for drug delivery through the application of an external magnetic field. Iron oxide is the most employed core because it exhibits high physiological stability and is easily removed through the endogenous iron metabolic pathway [75]. On account of their small size, MNPs can easily penetrate the brain matrix by temporarily creating pores in the BBB endothelium. The size and magnetic properties of synthesized MNPs are dependent on the physiological characteristics of the targeted organ [76]. Including their magnetic properties, the attractive characteristics of MNPs which include biocompatibility, low toxicity, easily modifiable surfaces have sprouted interest in drug delivery research [77]. Furthermore, since they can bind to several compounds such as drugs, antibodies and proteins, they can be directed to different receptors using an external magnetic field [77]. Despite mounting in vitro and in vivo data that indicate the potential applications of MNPs and other nano formulations, only a very few clinical trials have assessed their efficacy and safety on CNS conditions such as depression [77]. A study that was done using iron oxide nanoparticles proved that they are biocompatible and highly biodegradable under in vivo conditions. Interestingly, after metabolism, the iron can easily be incorporated into erythrocytes to form a part of hemoglobin, making it an added advantage [75]. In vivo studies that were done using rats to investigate the effects of iron oxide nanoparticles on depression treatment indicated that iron oxide nanoparticles are beneficial in reducing the symptoms of depression [78].

In another study, paroxetine and duloxetine-loaded nanogels were formulated to investigate the effect of MNPs on the efficacy of the antidepressants. The study showed that MNPs enhanced the release of the antidepressant. Magnetic fields induced stress on the nanoparticles, and this resulted in improved swelling properties of the nanogel. The group concluded that the use of magnetic nanoparticles could enhance the drug loading capacity and the sustained release profile of the formulation [46]. A previous study displayed that MNPs might be cleared by macrophages before reaching the targeted receptor or organ and the nanoparticles tend to aggregate due to strong magnetic interactions [77] which might result in increased toxicity and tissue damage. Moreover, in the absence of surface coating, the MNPs are prone to oxidation which may lead to the loss of magnetic field properties. However, aggregation can be prevented by coating the MNP with biopolymers, such as PEG and chitosan, which stabilize the nanoparticles. This might result in a reduction of antiparticle surface interaction [79]. Summary of nanocarriers discussed is presented in Table 2.

## 4. Surface Modification of Nanoparticles for Targeted Delivery of Antidepressants 

**Figure 5:**
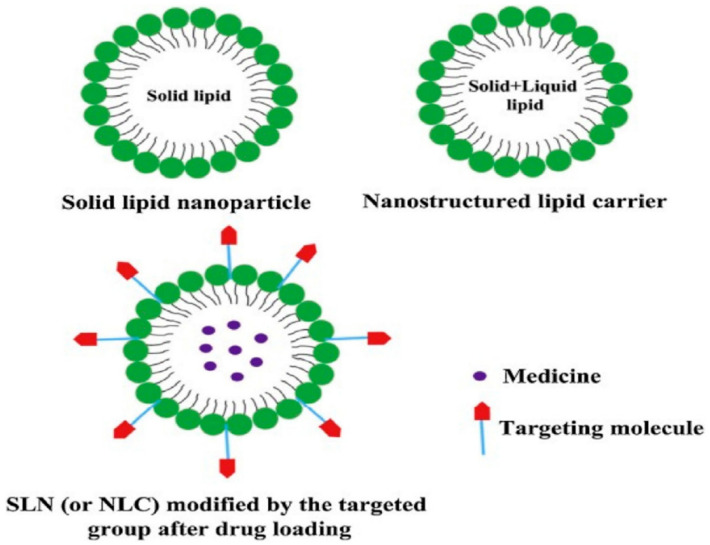
Surface modification of solid lipid nanoparticle. Modified SLN after conjugation of nanoparticles with ligands, to improve drug specificity and stability. The oral and neurobioavailability are improved and the nanoparticles exhibit a sustained release profile. Adapted from [87] Copyright (2019) Science Direct.

Nanotechnology systems have gained considerable attention over the past years due to their attractive properties which include a high surface-area-to-volume ratio [80,81]. However, nanoparticles possess limitations such as instability when exposed to biological fluids and lack of specificity [80]. There is therefore a pressing need to modify the nanoparticle-based drug delivery systems to improve drug efficacy. Surface modification of nanoparticles has gained vast attention due to the important properties conferred on the particles which include improved drug specificity, nanoparticle circulation time, safety, biocompatibility and solubility of hydrophobic drugs [82,83,84]. Most nanoparticles that are prepared from hydrophobic polymers are hydrophobic by default [85]. Hence, it is important for them to be modified accordingly and targeted to the required sites. This can be done by coating the nanoparticles with biopolymers such as PEG and complexing of them with ligands such as transferrin [86]. The coating of nanoparticles stabilizes them with no significant change on particle size). In addition, the coating of nanoparticles with hydrophilic polymers improves the circulation time, while the conjugation of nanoparticles with a ligand also improves the specificity of antidepressants (Figure 5) [85,87], thereby improving the bioavailability and efficacy of the antidepressants.

### Use Of Ligands to Improve the Specificity of Antidepressants and to Enhance Neuro Bioavailability

To achieve an optimal therapeutic outcome from an antidepressant drug delivery system, several factors should be considered. These include the solubility of the drug in physiological fluids, target specificity, the molecular weight of the drug and its particle size [88]. A limited concentration of antidepressants bind to targeted receptors; hence, employing targeting ligands such as transferrin, Apolipoprotein-E (Apo-E) and angiopep-2 can improve the potency and therapeutic outcome, by targeting the desired receptors and reducing the dose [89]. Administering a lower dosage might reduce the side effects while receptor-targeted delivery could enhance bioavailability in the targeted organs. Ligand-complexed drugs enable the recognition and targeting of specific receptors of the BBB. Consequently, this results in optimal drug delivery and prevents the drug from harming healthy tissues or inadvertently targeting other receptors. Moreover, due to the large surface area to volume ratio of nanoparticles, multiple ligands can be complexed in a process known as multivalent functionalization, thereby improving the binding affinity [89]. Moreover, efficient ligands for targeted drug delivery should have high binding infinity for the targeted receptors and the ability to penetrate to the targeted site [78]. For example, angiopep-2, can easily penetrate the BBB and has a high affinity for brain cells. A study by Masserini showed that complexing Angiopep-2 to nanoparticles coated with PEG caused a high accumulation of the drug delivered into the brain matrix [89,90]. 

Also, for effective antidepressant drug delivery and desired therapeutic outcome, the intrinsic drug needs to exceed the minimum threshold. Hence, an adequate number of ligands need to be complexed to the drug so that they can bind to sufficient receptors to enable receptor-mediated drug delivery to exceed the minimum threshold within a short period. This improves the therapeutic onset of the antidepressant and reduces its toxicity [7].

## 5. Toxicity of Nano-Based Antidepressants

As discussed above, nanotechnology offers a remarkable potential to improve the therapeutic effect of antidepressants. Nevertheless, nano-drug delivery systems have been reported in several studies to cause neurotoxicity [73]. For example, in vitro studies and clinical trials indicated that nanoparticles exhibit minor or major toxicity effects depending on the degree of toxicity of the biopolymers [73]. In an independent study, the degradation of other biopolymers such as PLGA could also induce cell damage due to the generation of an acidic medium which was dependent on the amount of polymer administered [35,91]. Another study showed that drug burst release of some oral bioactive agents from SLN nanocarriers might result in toxicity [15]. To assess the impact of nanoparticles on healthy brain cells, Teleanu et al., in 2018, showed that nanoparticles tend to accumulate in specific brain regions accessible to neural cells, such as neurons and microglia, leading to neurotoxicity and cell damage. Furthermore, iron oxide and gold nanoparticles have been reported as potential neurotoxic materials. For example, daily exposure to iron oxide nanoparticles can impair nerve conduction and synaptic transmission, resulting in inflammation and neural apoptosis [92]. Neural inflammation may breakdown the BBB, which may result in cerebral edema and nerve cell dysfunction [93]. Hence, it stands to reason that more studies need to be done to improve the safety of nanomedicines.

## 6. Conclusions and Future Perspectives

The use of nano-based drug delivery platforms has garnered considerable interest in the treatment of depression [80,94]. This review has summarized different nanocarriers and the route of administration that can be used to improve the efficacy and safety of the delivery of antidepressants. Currently prescribed antidepressants exhibit adverse effects such as delayed therapeutic onset, low bioavailability, and undesirable side effects. Nano-based drug delivery strategies possess attractive attributes to overcome the afore-mentioned limitations. These include sustained drug release, high drug specificity, increased bioavailability of the drug, improved drug absorption rate due to mucoadhesive properties of the biopolymers, enhanced drug delivery, systematic enhancement and low drug cytotoxicity [69]. Biopolymers protect drugs from undesirable phenomena which include the first-pass effect and harsh stomach conditions and can allow the permeation of the drug through the BBB due to their sizes and other properties. A critical appraisal of scientific literature has shown that the use of ligands enhances drug delivery by improving the drug concentration at the targeted receptors or organs. By 2016, 51 nanomedicines for various conditions were FDA approved and 77 were under clinical trials for other disease conditions [95]. In vitro and in vivo studies that were done using nanomedicines demonstrated positive outcomes in the treatment of depression, though more in vivo studies still need to be done to ensure the safety of the biopolymers and the nanocarriers [69]. Nanocarriers have the potential of increasing the therapeutic index hence enhancing drug efficacy. To our best knowledge, no nanomedicines have been approved for the treatment of depression to date. Nevertheless, several studies have shown that nanomedicines may improve the therapeutic efficacy of antidepressants. These observations warrant further research and clinical translation of nanomedicines against depression. Moreover, the safety of nanomedicines needs to be considered especially when they are prescribed for chronic diseases. This would ensure the complete degradation and nontoxicity of the biopolymers.

## Figures and Tables

**Table 1:** Summary of the application of biopolymers using different routes of administration.

Biopolymers	Benefits	Disadvantages	Drug	References
Oral				
sodium alginate HMC	Sustained release from microcapsules due to swelling properties of sodium alginate/hydroxypropyl methylcellulose copolymers at pH 7.4 and improved bioavailability due to mucoadhesive properties of sodium alginate when they bind to the epithelial mucous membrane lining.	Drug release is dependent on the concentration of the biopolymers	Venlafaxine	[14]
Sodium alginate	Increased circulation period enabled due to the mucoadhesive properties of sodium alginate which allows a delay in gastric emptying.	Solubility of alginate is dependent on pH of the solvent	Imipramine	[17]
Chitosan	Improved sustained release and circulation. Enhanced permeation due to mucoadhesive properties of chitosan which allows the nanoparticles to bind with the mucosa via the ionic interaction	Solubility of chitosan is affected by pH.	escitalopram	[18]
Chitosan-Arabinoxylan	Improved entrapping rate due to the swelling properties of the biopolymers. Sustained release from the microspheres due to swelling properties of chitosan/ arabinoxylan copolymer under acidic conditions of pH 1.2, due to protonation of the free amine groups on the copolymers	Encapsulation rate is directly proportional to the concentration of chitosan	Fluoxetine HCL	[20]
PEG-PLGA	Improved circulation, half-life and bioavailability due to amphiphilic copolymers of PEG-PLGA.	PLGA is not stable on its own	Dapoxetine	[25]
Intranasal				
Chitosan-PLGA	Sustained release profile due to hydration and swelling properties of CN/PLGA copolymers. Enhanced drug uptake rate and bioavailability as a result mucoadhesive and cationic properties of chitosan which increases the retention time of the nanoparticles in the nasal passage	PLGA cannot be stabilized by chitosan on its own	Desvenlafaxine	[27]
Chitosan sodium TPP	Amplified drug intranasal uptake and bioavailability as a result of mucoadhesive properties of chitosan and the interaction of cationic charges on the chitosan and anionic charges on the tight junction of the mucosal epithelium cells	Solubility of chitosan is affected by pH	Venlafaxine	[29]
Alginate	Higher mucoadhesive properties and permeation and sustained release. Enhanced therapeutic efficacy.	Covalent cross linking can result in cell toxicity	Venlafaxine	[30]
Nanostructured lipids	Increased drug release and drug efficacy due to improved residential time of the nanoparticles in the nasal cavity due to HPMC biopolymer.	Requires a stabilizer	Venlafaxine	[31]
Parenteral				
Chitosan	Improved half-life, entrapping rate and bioavailability owing it to mucoadhesive, encapsulation efficacy and delayed clearance properties of chitosan	Solubility of chitosan is affected by pH.	Sertraline	[21,33]
Polycaprolactone	Enhanced entrapping efficiency and sustained release.	More efficient with hydrophobic drugs Requires a stabilizer	L–tyrosine	[34]

**Table 2:** Summary of nanocarriers.

Type of Nanocarrier	Drug Delivery Characteristics	Structure	Drawbacks	References
Dendrimers	Rapid cellular entry, high drug loading capacity, improved half-life, biocompatibility	Highly branched, Monodisperse structure,	Non-degradable in physiological environment, Large particle size	[12,40]
Nanogels	Large surface area, high entrapping rate, biocompatible, high loading capacity,	Hydrogels, cross-linked hydrophilic polymer networks,	Physically cross-linked nanogels are less stable	[42,43]
Polymeric micelles	Increased half-life, solubility and stability, biodegradable, biocompatible	Amphiphilic Block copolymers,	Low drug loading capacity, Premature leaking,	[48,49,50,72]
Nanoliposomes	Enhanced encapsulating rate, biocompatible, biodegradable, improved intracellular uptake	Lipid vesicles, amphiphilic phospholipids	poor stability in aqueous	[53,55]
Carbon nanotubes	Improved cell-penetrating ability, biocompatibility, high drug entrapping rate,	Tubular morphology, two or more layers, allotropes of carbon	Mechanism is not known, too small, low solubility, permeability can be affected with temperature	[6,62]
Solid Lipid Nanoparticles	Excellent drug release profile, stable, biodegradable, large surface area	Spherical structure,	Poor incorporation rate, prone to gelation, loading capacity depends on length of the hydrocarbon chain,	[15,66]
Polymeric nanoparticles	High cell-penetrating rate, prolong duration, biodegradable, enhanced stability,	Spherical shape,	Easily eliminated in the bloodstream	[23,73]
Magnetic nanoparticles	High stability, biocompatible, improve drug targeting	Spherical structure, crystals.	Easily eliminated from the body, prone to aggregation	[75,77]
